# Benefit of primary and secondary prophylactic implantable cardioverter defibrillator in elderly patients

**DOI:** 10.1002/clc.24191

**Published:** 2023-11-14

**Authors:** Marie Lewenhardt, Fabienne Kreimer, Assem Aweimer, Andreas Pflaumbaum, Andreas Mügge, Michael Gotzmann

**Affiliations:** ^1^ University Hospital St Josef‐Hospital Bochum, Cardiology and Rhythmology Ruhr University Bochum Germany; ^2^ University Hospital Bergmannsheil Bochum, Cardiology Ruhr University Bochum Germany

**Keywords:** appropriate ICD therapy, benefit, elderly patients, implantable cardioverter defibrillator

## Abstract

**Background:**

The benefit of implantable cardioverter‐defibrillator (ICD) in elderly patients has been questioned. In the present study, we aimed to analyse the outcome of patients of different age groups with ICD implantation.

**Methods:**

We included all patients who received an ICD in our hospital from 2011 to 2020. Primary endpoints were (1) death from any cause and (2) appropriate ICD therapy (antitachycardia pacing/shock). A “benefit of ICD implantation” was defined as appropriate ICD therapy before death from any cause/or survival. “No benefit of ICD implantation” was defined as death from any cause without prior appropriate ICD therapy.

**Results:**

A total of 422 patients received an ICD (primary prophylaxis *n* = 323, secondary prophylaxis *n* = 99). At the time of implantation, 35 patients (8%) were >80 years and 106 patients were >75 years (25%). During the study period of 4.2 ± 3 years, benefit of ICD occurred in 89 patients (21%) and no benefit in 84 patients (20%). In primary prevention, the proportion of patients who had a benefit from ICD implantation decreased with increasing age, and there were no patients who benefited from ICD therapy in the group of patients >80 years. In secondary prophylaxis, the proportion of patients with a benefit from ICD implantation ranged from 20% to 30% in all age groups.

**Conclusion:**

Our study suggests that the indication of primary prophylactic ICD in elderly and very old patients should be critically assessed. On the other hand, no patient should be denied secondary prophylactic ICD implantation because of age.

## Graphical abstract

In primary prevention, the proportion of patients who had a benefit from implantable cardioverter‐defibrillator (ICD) implantation decreased with increasing age, and there were no patients who benefited from ICD therapy in the group of patients >80 years. Our study suggests that the indication of primary prophylactic ICD in elderly and very old patients should be critically assessed. However, our study suggest that no patient should be denied secondary prophylactic ICD implantation because of age.

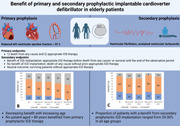

## 1 INTRODUCTION

For more than 20 years, implantation of an implantable cardioverter‐defibrillator (ICD) has been an established therapy for the prevention of sudden cardiac death, both in primary and secondary prevention.^1^, ^2^, ^3^, ^4^, ^5^ Based on these positive results, the European and American guidelines recommend the treatment with an ICD for a wide range of indications.^6^, ^7^ Implantation of the device has become routine surgery and is performed in approximately 100 000 patients/year in the European Union.^8^

Although the landmark studies^1^, ^2^, ^3^, ^4^, ^5^ demonstrated a prognostic advantage for the respective overall study population, certain subgroups had inconsistent outcomes. These subgroups consisted of patients with diabetes mellitus, patients with end‐stage renal disease, patients with nonischemic cardiomyopathy, and especially elderly and old patients.^5^, ^8^, ^9^, ^10^, ^11^, ^12^, ^13^ The question remains which patient may benefit from ICD implantation. This is an issue of major clinical importance because ICD implantation may be accompanied by periprocedural risk and the risk of long‐term adverse effects, which could be avoidable with a suitable selection of patients.

Because current guidelines clearly recommend ICD implantation,^6^, ^7^ a randomized trial to assess the improvement in prognosis with an ICD is problematic due to ethical concerns. However, benefit from ICD implantation can also be assumed if appropriate ICD therapy is performed to treat fatal ventricular tachycardia or ventricular fibrillation. In contrast, no benefit of ICD implantation can be assumed if patients die without ever receiving an appropriate ICD therapy.

The aim of the present study was to investigate the characteristics of patients with and without benefit of ICD implantation, focusing on the analysis of different age groups, especially elderly and very old patients. A further comparison was undertaken between patients with primary or secondary prophylactic ICD indication. The aim was to identify subgroups of patients in whom no benefit of ICD implantation is detectable.

## 2 METHODS

### 2.1 Inclusion and exclusion criteria

This retrospective study examined all patients who received an ICD between 2011 and 2020 at St. Josef Hospital Bochum. Patients in whom ICD interrogation could not be followed up after hospital discharge were excluded from the analysis. All types of ICD therapy were included in the analysis: endovascular and subcutaneous ICD as well as ICD with and without cardiac resynchronization therapy. In addition, both patients with first implantation and patients with generator replacement were included in this study. ICD implantation was for primary prophylactic or secondary prophylactic indication, according to the current guidelines at the time of implantation.^14^, ^15^ The study was approved by the local ethics committee of the Ruhr University Bochum (Number 21‐7439‐BR).

### 2.2 Study endpoints

Primary endpoints of the study were (1) death from any cause and (2) appropriate ICD therapy (appropriate antitachycardia pacing [ATP] and/or appropriate ICD shock due to ventricular fibrillation or sustained ventricular tachycardia).

Secondary endpoint was “benefit of ICD implantation.” A “benefit of ICD implantation” was defined as appropriate ICD therapy before death from any cause/or survival until the end of the observation period. “No benefit of ICD implantation” was defined as death of any cause without prior appropriate ICD therapy. A neutral outcome was present in surviving patients without appropriate ICD therapy.

### 2.3 Data acquisition and follow‐up

The patients' data were collected by the medical history including medication, laboratory results, ECG, and echocardiography at the time of ICD implantation.

All patients were consulted in our outpatient clinic for a routine check‐up and device interrogation 6 weeks after implantation. Thereafter, the ICD function was checked regularly at 6‐month intervals. These visits were performed either at our university outpatient clinic or at the cardiologist's practice. For the assessment of ICD shocks and ATP, documentation of all interrogations of the patients' ICD was examined. The analysis of the study was based on the first appropriate ICD therapy and the corresponding arrhythmia. In addition, inappropriate ICD therapy and other complications of device therapy were recorded.

For the analysis of survival, a follow‐up was performed between November 2021 and April 2022. For this purpose, the data of the routine examinations at our university outpatient clinic and a telephone contact with the patients were used. In case of deceased patients, contact was conducted with the patients' primary care physicians.

### 2.4 Statistics

The statistical software SPSS 26 was used for statistical analysis. Numerical values are expressed as mean ± standard deviation. Continuous variables were compared between groups using an unpaired *t* test (for normally distributed variables) or Mann–Whitney *U* test (for nonnormally distributed variables) or Kruskal–Wallis test (for nonnormally distributed variables), when appropriate. *χ* ^2^ analysis was used to compare categoric variables. Receiver operating characteristic analysis was used to determine cut‐off values for discriminating patients with benefit from ICD implantation versus patients without benefit from ICD implantation/neutral outcome with respect to age at the time of implantation.

Survival and event‐free survival (freedom from appropriate ICD therapy) in different age groups were analyzed by the Kaplan–Meier method, and curves were compared by the log‐rank test. A *p* < 0.05 was considered significant. All probability values reported are 2‐sided.

## 3 RESULTS

In total, 437 patients received an ICD at the university hospital St Josef Hospital between 2011 and 2020. A total of 15 patients were lost in follow‐up (3%). In absence of any information, these patients were excluded from the analysis.

### 3.1 Patient groups

Of the remaining 422 patients, 335 underwent first‐time implantation of an ICD. In 102 patients, an ICD generator was replaced or an already implanted ICD system was upgraded to cardiac resynchronization with ICD (CRT‐D).

CRT‐D implantation was performed in 31% of all patients (*n* = 134). A total of 14 patients received a subcutaneous ICD (3%). The implanted ICD devices were manufactured by Boston Scientific/Guidant/Cameron health, Biotronik, Medtronic, and St. Jude Medical/Abbott.

There was a primary prophylactic indication for ICD implantation in 323 patients (77%). Of these patients, 164 patients (51%) had ischemic cardiomyopathy and 148 patients (46%) had nonischemic cardiomyopathy. A total of 11 patients (3%) underwent primary prophylactic ICD implantation because of other causes (such as hypertrophic cardiomyopathy with or without obstruction or long‐QT syndrome).

In 99 patients (23%), there was a secondary prophylactic indication for ICD implantation. Here, the indications leading to ICD implantation were ventricular fibrillation in 65 patients (66%) and sustained ventricular tachycardia in 34 patients (34%).

### 3.2 Patient characteristics

Of the 422 patients, 86 were women (20%). Mean left ventricular ejection fraction was 32.3 ± 10.3% (ranged from 15% to 65%). Patients had a mean age of 66.9 ± 11.3 years (ranged from 22 to 89 years). Clinical characteristics at the time of ICD implantation are given in Tables 1 and 2.

**Table 1:** Clinical characteristics of study patients (*n* = 422).

	Total study cohort (*n* = 422)	Group A = benefit (*n* = 89)	Group B = no benefit (*n* = 84)	Group C = neutral (*n* = 249)	*p* Value
Age (years)	66.9 ± 11.4	64.2 ± 11.9*	72.9 ± 6.88***	65.8 ± 11.7	<0.001
Women (♀), *n* (%)	86 (20)	15 (17)	17 (20)	54 (22)	0.623
**Indication for ICD**					
Primary prevention, *n* (%)	323 (77)	59 (66)***	72 (86)	192 (77)	0.010
Secondary prevention, *n* (%)	99 (23)	30 (34)***	12 (14)	57 (23)	0.010
Cardiac resynchronization therapy, *n* (%)	134 (32)	18 (20)***	30 (36)	86 (35)	0.031
Generator replacement, *n* (%)	102 (24)	27 (30)	19 (23)	56 (22)	0.159
Inadequate ICD shock, *n* (%)	30 (7)	6 (7)	6 (7)	18 (7)	0.988
**Medical history**					
Hypertension, *n* (%)	316 (75)	61 (69)	68 (81)	187 (75)	0.169
Dyslipidemia, *n* (%)	209 (50)	46 (52)	44 (52)	119 (48)	0.691
Diabetes mellitus, *n* (%)	148 (35)	24 (27)*	39 (44)***	85 (34)	0.024
Coronary artery disease, *n* (%)	241 (57)	53 (60)	48 (57)	140 (56)	0.894
Myocardial infarction, *n* (%)	161 (38)	44 (49)**	32 (38)	85 (34)	0.039
Coronary artery bypass grafting, *n* (%)	78 (18)	17 (19)	19 (23)	42 (17)	0.270
Atrial fibrillation, *n* (%)	148 (35)	32 (36)	36 (43)	80 (32)	0.183
Stroke and/or TIA, *n* (%)	73 (17)	14 (16)	18 (21)	41 (16)	0.529
Chronic obstructive lung disease, *n* (%)	63 (15)	15 (17)	20 (24)***	28 (11)	0.017
Peripheral artery disease, *n* (%)	74 (18)	12 (13)	21 (25)	41 (16)	0.108
**Medication**					
ACEI or ARB or ARNI, *n* (%)	380 (90)	80 (90)	76 (90)	224 (90)	0.760
Betablocker, *n* (%)	378 (90)	78 (88)	78 (93)	222 (89)	0.399
Loop diuretics, *n* (%)	287 (68)	50 (56)*	69 (82)***	168 (67)	<0.001
Aldosterone antagonist, *n* (%)	259 (61)	49 (55)	45 (54)***	165 (66)	0.056
Amiodarone, *n* (%)	61 (14)	14 (16)	17 (20)	30 (12)	0.154

Abbreviations: ACEI or ARB or ARNI, angiotensin‐converting enzyme inhibitor or angiotensin receptor blocker angiotensin receptor‐neprilysin inhibitor; ICD, implantable cardioverter defibrillator; TIA, transient ischemic attack.

* Group A versus Group B (*p* < 0.05).

** Group A versus Group C (*p* < 0.05).

*** Group B versus Group C (*p* < 0.05).

**Table 2:** Echocardiographic, electrocardiographic, and laboratory characteristics of study patients (*n* = 422).

	Total study cohort (*n* = 422)	Group A = benefit (*n* = 89)	Group B = no benefit (*n* = 84)	Group C = neutral (*n* = 249)	*p* Value
**Echocardiography**					
Left atrial diameter (mm)	43.5 ± 6.58	43 ± 6.28*	45.6 ± 5.61***	43.1 ± 6.83	0.021
Left ventricular ejection fraction (%)	32.4 ± 10.3	34 ± 10.8	32 ± 9.03	32 ± 10.4	0.234
**Electrocardiography**					
Heart rate (beats/min)	75.7 ± 19.7	74 ± 19.4	75 ± 16.1	76.6 ± 20.8	0.713
Sinus rhythm, *n* (%)	334 (79)	71 (80)	58 (69)***	205 (82)	0.070
Left bundle branch block, *n* (%)	117 (28)	20 (22)	19 (23)	78 (31)	0.140
**Labor**					
Hemoglobin (g/dL)	13.3 ± 1.89	13.6 ± 1.97*	12.7 ± 1.75***	13.4 ± 1.86	<0.001
Creatinine (mg/dL)	1.18 ± 0.38	1.13 ± 0.34*	1.31 ± 0.43***	1.15 ± 0.37	<0.001

* Group A versus Group B (*p* < 0.05).

** Group A versus Group C (*p* < 0.05).

*** Group B versus Group C (*p* < .05).

At the time of implantation, 35 patients (8%) were >80 years, 106 patients (25%) were >75 years, 182 patients (43%) were >70 years, 255 patients (60%) were >65 years, and 167 patients were ≤65 years (40%).

### 3.3 Primary study endpoints: all‐cause death & ICD therapy

The mean follow‐up was 4.2 ± 3.0 years. Of the 422 study patients, 106 patients died (25%). Of these patients, 40 died of cardiovascular death (cardiac pump failure, *n* = 23; ventricular tachycardia/ventricular fibrillation, *n* = 6; myocardial infarction, *n* = 4; cardiogenic sepsis, *n* = 3 [device‐associated, *n* = 1; Mitral valve endocarditis, *n* = 1; left ventricular assist device infection, *n* = 1]; vascular surgery, *n* = 2; pulmonary artery embolism, *n* = 1; other, *n* = 1). A total of 40 patients died of noncardiovascular causes (carcinoma, *n* = 11; pneumonia, *n* = 9; sepsis, *n* = 7; trauma surgery causes, *n* = 4; suicide *n* = 1; other *n* = 8). No exact cause of death could be determined in 26 patients. In the group of patients >80 years of age, 31% (11 of 35 patients) died during the study period. Of the 11 patients, four died from cardiovascular causes, three from noncardiovascular causes, and four patients from uncertain causes.

A total of 90 patients (21%) received appropriate ICD therapy. First appropriate ICD therapy occurred at a mean of 804 ± 852 days (ranged from 1 to 3260 days) after implantation. The reasons of first appropriate ICD therapy were sustained ventricular tachycardia in 71 patients and ventricular fibrillation in 19 patients. These arrhythmias were terminated with ATP in 31 patients and with ICD shock in 59 patients (and some previously with unsuccessful ATP).

Of the 90 patients with appropriate ICD therapy, 22 patients (24%) died during study period. The mean time between appropriate ICD therapy and subsequent death of these 22 patients was 765 ± 674 days (ranged from 39 to 2302 days). One patient received appropriate ICD therapy but died on the same day due to cardiogenic shock and multiorgan failure. No benefit of ICD implantation was observed in this patient.

### 3.4 Secondary endpoints—Benefit of ICD implantation

Benefit of ICD implantation (defined as appropriate ICD therapy before death from any cause/or survival until the end of the observation period) occurred in 89 patients (21%). No benefit of ICD implantation (defined as death of any cause without prior appropriate ICD therapy) was seen in 84 patients (20%). A neutral outcome was present in 249 surviving patients (59%) without appropriate ICD therapy.

Patients with a benefit of ICD implantation were younger, more likely to have a secondary prophylactic ICD indication, and less likely to have cardiac resynchronization therapy compared with patients without a benefit of ICD implantation. The differences in patient characteristics in the three groups are presented in Tables 1 and 2. The distribution of age groups is presented in Table 3.

**Table 3:** Age distribution of patients (*n* = 422).

	Total study cohort (*n* = 422)	Group A = benefit (*n* = 89)	Group B = no benefit (*n* = 84)	Group C = neutral (*n* = 249)	*p* Value
**Age subgroups**					<0.001
Age ≤65 years, *n* (%)	167 (40)	43 (48)	11 (13)	113 (45)	
Age 66–70 years, *n* (%)	73 (17)	17 (19)	19 (23)	37 (15)	
Age 71–75 years, *n* (%)	76 (18)	16 (18)	22 (26)	38 (15)	
Age 76–80 years, *n* (%)	71 (17)	10 (11)	21 (25)	40 (16)	
Age >80 years, *n* (%)	35 (8)	3 (3)	11 (13)	21 (8)	

**Figure 1:**
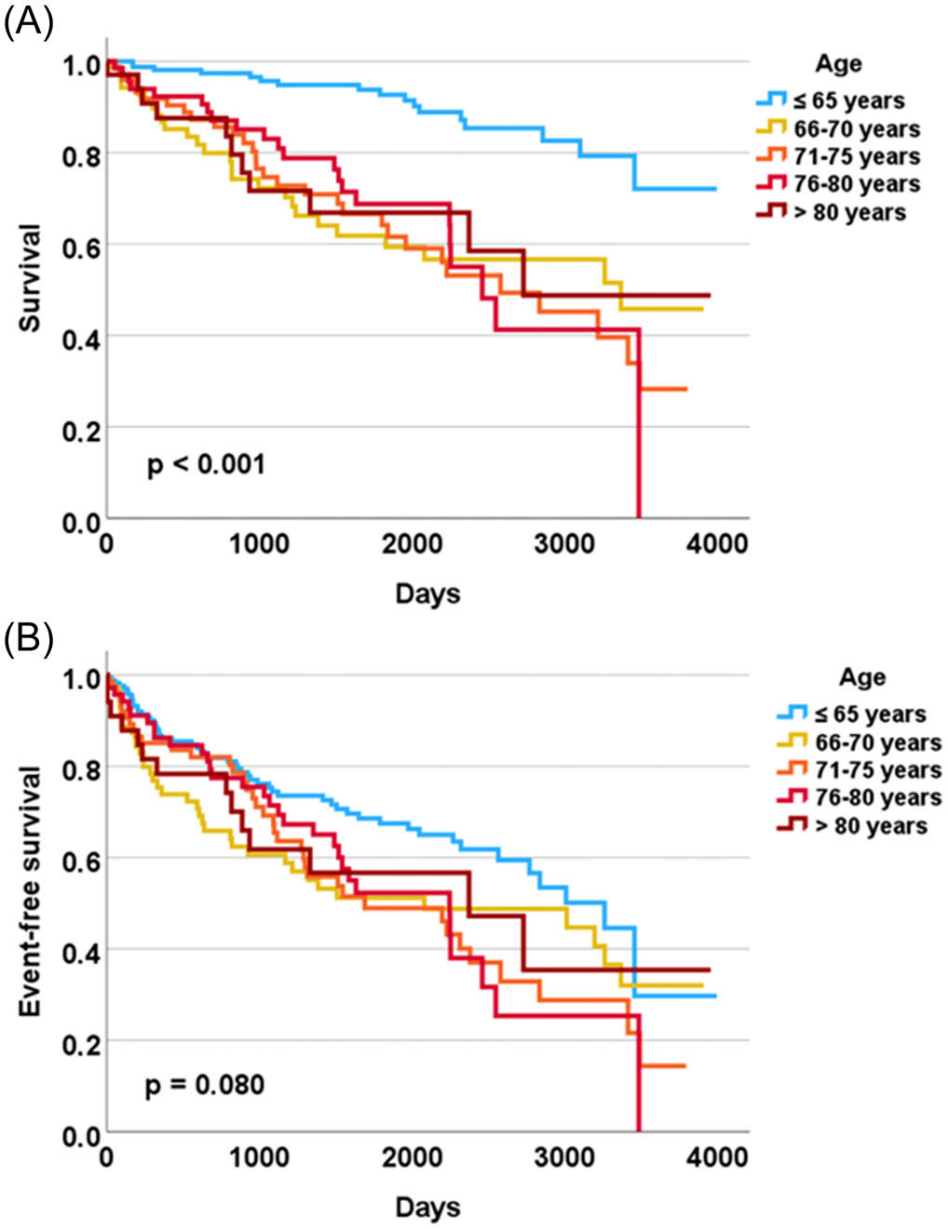
Kaplan–Meier estimates the survival (A) and the event‐free survival (combined end point of death and appropriate implantable cardioverter‐defibrillator shock) (B) in different age groups of study patients (*n* = 422).

While all‐cause mortality demonstrated an association with patient age, the combined endpoint (all‐cause death & appropriate ICD therapy) did not differ significantly between age groups. The Kaplan–Meier curves for the entire study cohort are presented in Figure 1.

Receiver operating characteristic analysis (age in relation to benefit of ICD implantation) demonstrated an area under the curve of 0.587 (confidence interval 0.523–0.651, *p* = 0.008). The best cut‐off value for the benefit of ICD implantation was an age of <66 years, but with low sensitivity and specificity (53% and 61%, respectively).

Inappropriate ICD therapy occurred in 30 patients (7%). In 20 patients this was due to supraventricular tachycardia (mainly in tachycardic atrial fibrillation) and in 10 patients due to shock coil failure (lead fracture or dislocation). Frequency of occurrence of inappropriate shock did not differ among the groups of patients (Table 1).

### 3.5 Subgroup analysis

We divided the study cohort in two subgroups depending on whether the patients received the ICD due to primary prophylaxis (*n* = 323, 77%) or due to secondary prophylaxis (*n* = 99, 23%).

### 3.6 Primary prophylaxis

**Figure 2:**
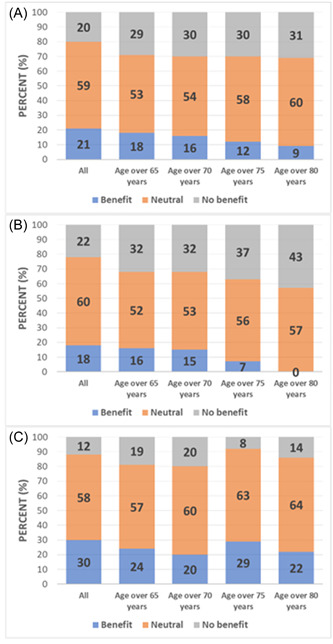
Frequency of outcomes at different ages in (A) all study patients (B) in the subgroup of patients with primary prophylactic implantable cardioverter‐defibrillator (ICD) indication and (C) in the subgroup of patients with secondary prophylactic ICD indication (Benefit of ICD therapy was defined as appropriate ICD therapy before death from any cause/or survival. No benefit of ICD therapy was defined as death of any cause without prior appropriate ICD therapy. A neutral outcome was present in surviving patients without ICD therapy).

Among patients with a primary prophylactic indication, 18% had a benefit of ICD implantation and 22% had no benefit. The proportion of patients with a benefit of ICD implantation decreased in the older age groups, whereas the proportion of patients without a benefit increased. In patients aged >80 years (*n* = 21), there were no patients who received appropriate ICD therapy. In contrast, a relevant proportion in this age group died without prior appropriate ICD therapy (Figure 2). Accordingly, the Kaplan–Meier curves presented significantly lower all‐cause mortality in patients ≤65 years of age compared with older patients, whereas event‐free survival (freedom from appropriate ICD therapy) revealed no significant difference in the different age groups (Figure S1). Patients with a primary prophylactic ICD indication and a benefit of ICD implantation were more likely to have a history of myocardial infarction, were less likely to receive loop diuretics, and had higher hemoglobin levels than patients without a benefit of ICD implantation (Table S1 and S2).

### 3.7 Secondary prophylaxis

In the subgroup of patients with secondary prophylactic indication, 30% had a benefit of ICD implantation. Only 12% of patients died without prior appropriate ICD therapy (Figure 2). The proportion of patients with a benefit of ICD implantation was slightly lower in the older age groups but amounted to 22% in the group of patients aged >80 years (*n* = 14) (Figure 2). Accordingly, the rates of event‐free survival were not significantly different in different age groups (Figure S2). Patients without a benefit of ICD implantation were significantly older, more likely to have diabetes mellitus, and more likely to have peripheral arterial disease than patients with benefit of ICD implantation (Tables S3 and S4).

### 3.8 Other subgroups

In our study, no significant difference in the rate of benefit of ICD implantation was detected between men and women or between patients who received an ICD for the first time and those who received a generator replacement (Table 1).

However, differences were detected in patients with cardiac resynchronization therapy versus those without cardiac resynchronization therapy (Table 1). Our study suggests that these patients have a lower benefit of ICD therapy (appropriate ICD therapy for life‐threatening arrhythmia). Since the mortality of patients with and without cardiac resynchronization therapy revealed no difference in the Kaplan‐Meier analysis (*p* = 0.623), this indicates that patients with cardiac resynchronization therapy received less appropriate ICD therapy. Therefore, this could rather indicate a positive effect of cardiac resynchronization therapy.

## 4 DISCUSSION

The present study investigated the benefit of ICD implantation in different subgroups of patients. Benefit of ICD implantation was defined as appropriate ICD therapy resulting in prolonged survival. In contrast, no benefit was assumed if patients died without a prior appropriate ICD therapy. In our study, which included all ICD implantations at St. Josef Hospital from 2011 to 2020, an age‐dependent benefit of ICD implantation was observed in the group of patients with primary prophylactic ICD indication.

The most important finding of the study is that in the group of patients over 80 years of age, no patients benefited from ICD implantation, which was partly due to high mortality. In contrast, all age groups in the secondary prophylactic ICD indication group demonstrated a relevant proportion of patients with a benefit of ICD implantation (Figure 2).

### 4.1 ICD implantation in elderly patients

Prognostic advantage of survival was demonstrated for ICD implantation both in primary and secondary prevention.^1^, ^2^, ^3^, ^4^, ^5^ However, the median age of patients included in the landmark trials was between 58 and 65 years.^1^, ^2^, ^3^, ^4^, ^5^ Due to the aging of the population and changes in the treatment of structural heart disease, much older patients present nowadays with an indication for ICD implantation. These elderly and very old patients have a high mortality rate due to both their heart disease and advanced age, which is not only due to ventricular arrhythmias. Therefore, in recent years, there has been a critical and controversial discussion about the benefit of ICD implantation in elderly and old patients.^5^, ^8^, ^9^, ^10^, ^11^, ^12^, ^13^ Nevertheless, current European and American guidelines recommend primary or secondary prophylactic ICD implantation regardless of patient age.^6^, ^7^

According to the current annual report of the German Defibrillator Registry, approximately 21 000 new ICD are implanted in Germany each year. Of these, approximately one‐third of patients receive ICD implantation due to a secondary prophylactic indication and two‐thirds receive ICD implantation due to a primary prophylactic indication. Although the benefit of ICD therapy in elderly and old patients is controversial, the proportion of patients ≥80 years in Germany is 14%,^16^ like previous surveys from the United States.^17^, ^18^

In our study, the mean age at the time of ICD implantation was 66.9 ± 11.3 years (22–89 years). Overall, 8% of the total cohort were >80 years and 25% were >75 years (Table 3). While overall survival was significantly different between age groups, event‐free survival (freedom from appropriate ICD therapy) demonstrated no difference between age groups. This means that the elderly and old patients died significantly earlier during the study period, whereas the younger patients (especially patients ≤65 years) had fewer deaths and more appropriate ICD therapies (Figure 1). Our study thus indicates that the benefit of ICD implantation decreases in older age, as the proportion of patients who die from other than arrhythmogenic causes increases significantly. In this regard, our study is consistent with recently published studies.^5^, ^8^, ^9^, ^10^, ^11^, ^12^, ^13^

### 4.2 Controversies about primary prophylactic ICD implantation

The DANISH trial included 1116 patients with symptomatic nonischemic heart failure and reduced left ventricular ejection fraction (≤35%). Patients were randomized 1:1 into a group of patients who had an ICD implanted and a group of patients without ICD implantation. This study demonstrated that in the overall cohort, primary prophylactic ICD implantation had no prognostic advantage of survival in patients with nonischemic cardiomyopathy.^13^ However, in the subgroup of patients younger than 68 years, the rate of death from any cause was significantly lower in the ICD group than in the control group.^13^

Although we cannot comment on prognostic effects of an ICD implantation in our study, our results nevertheless indicate that the benefit of ICD implantation in both primary and secondary prevention is greatest in younger patients (≤65 years) (Figures 1 and 2). In our study, the best age threshold for discriminating a benefit of ICD implantation was <66 years, but with insufficient sensitivity and specificity (53% and 61%, respectively).

Interestingly, in our study, there was no difference in the benefit of ICD therapy between patients with ischemic cardiomyopathy and nonischemic cardiomyopathy. However, patients with previous myocardial infarction were significantly more likely to have appropriate ICD therapy and to derive a higher proportion of benefit from ICD implantation (Table S1).

In the prospective study “EUropean Comparative Effectiveness Research to Assess the Use of Primary ProphylacTic Implantable Cardioverter‐Defibrillators (EU‐CERT‐ICD)”, it has been recently revealed that in patients with primary prophylactic ICD indication (both ischemic and nonischemic cardiomyopathy) there is a significant survival advantage in the group of patients with ICD.^8^ However, subgroup analysis showed that there was no advantage in patients with diabetes and in patients aged ≥75 years.^8^

Consistent with this, patients with diabetes mellitus were more likely to have no benefit from ICD implantation in our study (Table 1). This observation is in accordance with the results of a meta‐analysis that analyzed patient‐level data from four major randomized controlled trials of ICD.^19^

In our study, 25% of all patients were >75 years at the time of implantation. This proportion of patients is not unusual compared to other studies and registries (12, 17, 20 21). Of particular clinical significance is the finding in our study that in the group of patients with primary prophylactic ICD indications aged >80 years, no patient exhibited benefit from ICD implantation (Figure 2B).

### 4.3 Controversies about secondary prophylactic ICD implantation

Prospective randomized trials of secondary prophylactic implantation were published over 20 years ago.^2^, ^3^, ^20^ Compared with amiodarone therapy, there was a prognostic benefit, but the mean age of patients was 63.7 ± 10.4 years and less than 15% of patients were ≥75 years (Healey 11).

Pooled analysis of these three studies showed that patients aged ≥75 years had a high incidence of nonarrhythmic death, and a prognostic benefit of ICD implantation could not be demonstrated.^11^

Recently, data of an American register on patients who underwent ICD implantation for secondary prophylactic indications were published.^21^ Noteworthy was that the mean age of the patients at implantation was 75 years and that about a quarter of the patients were ≥80 years old. Although higher mortality was also observed in the elderly and very old patients, the authors noted that four of five patients were still alive 2 years after implantation, which may support the use of an ICD even in elderly patients.^21^

Similarly, age was recently found to be an independent predictor of death after ICD implantation for secondary prophylactic indications. However, mortality rates in patients ≥80 years did not differ from those aged 70–79 years.^22^ Of note, in contrast to prospective randomized trials conducted many years ago, the rate of elderly and very old patients increased significantly in routine clinical practice.

In our study, 14% of patients with secondary prophylactic ICD implantation were >80 years old and 25% were ≥75 years old. Our study could not comment on mortality. However, we demonstrated a relevant proportion of patients (20%–30%) in each age group who received appropriate ICD therapy (Figure 2C), SO we assume a clinical benefit of ICD implantation even in very old patients. Nevertheless, our study suggests that patients with peripheral artery disease and diabetes mellitus have a lower rate of benefit from ICD implantation (Table S3). This observation should possibly be considered in the individual decision to implant an ICD even at an advanced age.

## 5 LIMITATIONS

In our study, the benefit of ICD implantation was defined as appropriate ICD therapy (appropriate ICD shock or appropriate ATP). A statement on the impact on mortality was not possible because of a missing control group due to ethical concerns. The cause of death of some of the deceased patients could not be clearly identified. In addition, no ICD interrogation was performed after the death of a patient, so that more patients may have died of an arrhythmogenic death.

An important limitation of the study is that it is a retrospective and monocentric analysis. However, we included almost all patients who received an ICD in our hospital from 2011 to 2020. Only 3% of patients were lost to follow‐up. Thus, we have a good insight into a group of patients who underwent ICD implantation according to standard clinical recommendations.

Another important limitation is the relatively small group size, especially of patients older than 80 years. This is partly due to the more conservative procedure in patients of very old age—an approach that is supported by the results of the present study.

## 6 CONCLUSION

In our analysis of patients with primary prophylactic ICD implantation, there is a decreasing benefit of ICD therapy with increasing age. Our study suggests that the indication of primary prophylactic ICD in elderly and very old patients should be critically assessed.

On the other hand, in patients with secondary prophylactic ICD implantation, there was a benefit of ICD therapy across all age groups. The group of patients >75 years and even >80 years also revealed a benefit of ICD therapy. Our study suggests that no patient should be denied secondary prophylactic ICD implantation because of age.

## CONFLICT OF INTEREST STATEMENT

MG was a speaker for Abbott, Bristol‐Myers Squibb, Novartis, Pfizer, and an advisor for Boehringer Ingelheim. The other authors declare no conflict of interest.

## Supporting information


**Figure Supplement 1:** Kaplan‐Meier estimates the survival (A) and the event‐free survival (combined end point of death and appropriate ICD shock) (B) in in the subgroup of patients with primary prophylactic ICD indication (n = 323).

**Figure Supplement 2:** Kaplan‐Meier estimates the survival (A) and the event‐free survival (combined end point of death and appropriate ICD shock) (B) in in the subgroup of patients with secondary prophylactic ICD indication (n = 99).

Supporting information.

## Data Availability

Data available on request due to privacy/ethical restrictions. The data that support the findings of this study are available on request from the corresponding author. The data are not publicly available due to privacy or ethical restrictions.
