# Supplementary figures and images for: Benefit of primary and secondary prophylactic implantable cardioverter defibrillator in elderly patients
Source: Clin Cardiol. 2023 Nov 14;47(2):e24191. doi: 10.1002/clc.24191 (PMC10826786; doi:10.1002/clc.24191)

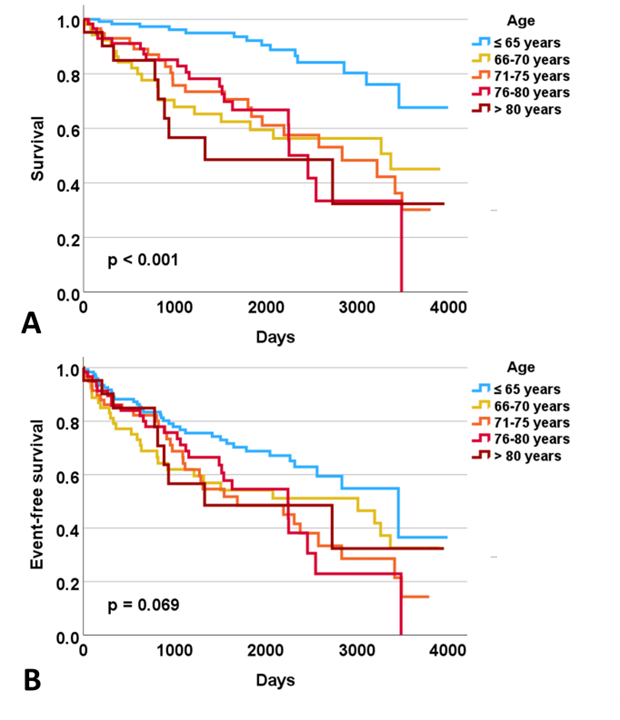

Supplement: Supplementary file 1 — Figure Supplement 1: Kaplan‐Meier estimates the survival (A) and the event‐free survival (combined end point of death and appropriate ICD shock) (B) in in the subgroup of patients with primary prophylactic ICD indication (n = 323). [file CLC-47-e24191-s003.jpg]

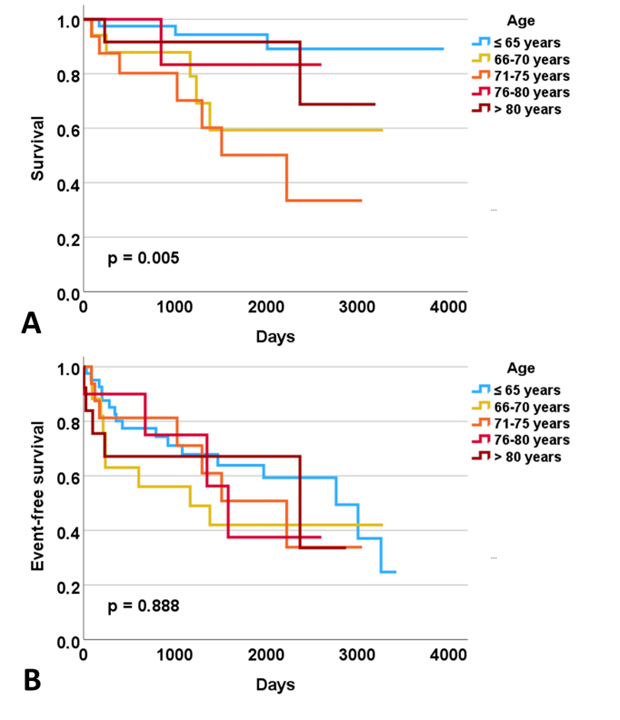

Supplement: Supplementary file 2 — Figure Supplement 2: Kaplan‐Meier estimates the survival (A) and the event‐free survival (combined end point of death and appropriate ICD shock) (B) in in the subgroup of patients with secondary prophylactic ICD indication (n = 99). [file CLC-47-e24191-s001.jpg]
