# Supplementary material for: Benefit of primary and secondary prophylactic implantable cardioverter defibrillator in elderly patients
Source: Clin Cardiol. 2023 Nov 14;47(2):e24191. doi: 10.1002/clc.24191 (PMC10826786; doi:10.1002/clc.24191)
Supplement: Supplementary file 3 — Supporting information. [file CLC-47-e24191-s002.docx]

**Table 1 Supplement 1** Clinical characteristics of patients with primary prophylaxis (n = 323)

|  | **Total study cohort (n = 323)** | **Group A = Benefit (n = 59)** | **Group B = No Benefit (n = 72)** | **Group C = Neutral (n = 192)** | **P value** |
| --- | --- | --- | --- | --- | --- |
| Age (years) | 67 ± 10.9 | 64.4 ± 11.2 ∗ | 72.8 ± 7 ‡ | 65.7 ± 11.4 | < 0.001 |
| Women (♀), n (%) | 65 (20) | 9 (15) | 15 (21) | 41 (21) | 0.585 |
| **Indication for ICD** |  |  |  |  |  |
| Ischemic cardiomyopathy | 164 (51) | 35 (59) | 30 (42) | 89 (46) | 0.143 |
| Non-ischemic DCM | 148 (46) | 22 (37) | 40 (55) | 96 (50) | 0.167 |
| Other reason | 11 (3) | 2 (4) | 2 (3) | 7 (4) | 0.942 |
| Cardiac resynchronisation therapy, n (%) | 127 (39) | 16 (27) † | 30 (42) | 81 (42) | 0.105 |
| Generator replacement, n (%) | 73 (23) | 13 (22) | 23 (32) | 37 (19) | 0.090 |
| Inadequate ICD shock, n (%) | 22 (7) | 3 (5) | 5 (7) | 14 (7) | 0.840 |
| **Medical history** |  |  |  |  |  |
| Hypertension, n (%) | 246 (76) | 41 (69) | 60 (83) | 145 (76) | 0.171 |
| Dyslipidemia, n (%) | 167 (52) | 34 (58) | 38 (53) | 95 (49) | 0.537 |
| Diabetes mellitus, n (%) | 121 (37) | 19 (32) | 31 (43) | 71 (37) | 0.432 |
| Coronary artery disease, n (%) | 179 (55) | 36 (61) | 40 (56) | 103 (54) | 0.628 |
| Myocardial infarction, n (%) | 113 (35) | 32 (54) ∗ † | 26 (36) | 55 (29) | 0.001 |
| Coronary artery bypass grafting, n (%) | 62 (19) | 12 (20) | 17 (24) | 33 (17) | 0.201 |
| Atrial fibrillation, n (%) | 112 (35) | 19 (32) | 32 (44) ‡ | 61 (32) | 0.125 |
| Stroke and/or TIA, n (%) | 53 (16) | 8 (14) | 14 (19) | 31 (16) | 0.656 |
| Chronic obstructive lung disease, n (%) | 54 (17) | 11 (19) | 18 (25) ‡ | 25 (13) | 0.061 |
| Peripheral artery disease, n (%) | 54 (17) | 8 (14) | 15 (21) | 31 (16) | 0.511 |
| **Medication** |  |  |  |  |  |
| ACEI or ARB or ARNI, n (%) | 301 (93) | 54 (92) | 66 (92) | 181 (94) | 0.943 |
| Betablocker, n (%) | 292 (90) | 52 (88) | 67 (93) | 173 (90) | 0.524 |
| Loop diuretics, n (%) | 239 (74) | 38 (64) ∗ | 63 (88) ‡ | 138 (72) | 0.005 |
| Aldosterone antagonist, n (%) | 225 (70) | 41 (69) | 41 (57) ‡ | 143 (74) | 0.031 |
| Amiodarone, n (%) | 40 (12) | 9 (15) | 13 (18) ‡ | 18 (9) | 0.111 |

ICD, implantable cardioverter defibrillator; TIA, transient ischaemic attack; ACEI or ARB or ARNI, angiotensin converting enzyme inhibitor or angiotensin receptor blocker angiotensin receptor-neprilysin inhibitor.

∗ Group A vs. Group B (p < 0.05), † Group A vs. Group C (p < 0.05), ‡ Group B vs. Group C (p < 0.05).

**Table Supplements 2** Echocardiographic, electrocardiographic and laboratory characteristics of patients with primary prophylaxis (n = 323)

|  | **Total study cohort (n = 323)** | **Group A = Benefit (n = 59)** | **Group B = No Benefit (n = 72)** | **Group C = Neutral (n = 192)** | **P value** |
| --- | --- | --- | --- | --- | --- |
| **Echocardiography** |  |  |  |  |  |
| Left atrial diameter (mm) | 44 ± 6.4 | 43.7 ± 6.55 | 45.9 ± 5.48 ‡ | 43.6 ± 6.58 | 0.048 |
| Left ventricular ejection fraction (%) | 29.8 ± 7.4 | 31 ± 7.64 | 31 ± 7.51 | 29.1 ± 7.25 | 0.123 |
| **Electrocardiography** |  |  |  |  |  |
| Heart rate (beats/min) | 76.1 ± 18.9 | 74.4 ± 19.5 | 74.7 ± 14.4 | 77.1 ± 20.1 | 0.909 |
| Sinus rhythm, n (%) | 256 (79) | 47 (80) | 51 (71) | 158 (82) | 0.188 |
| Left bundle branch block, n (%) | 102 (32) | 15 (25) | 19 (26) | 68 (35) | 0.198 |
| **Labor** |  |  |  |  |  |
| Hemoglobin (g/dL) | 13.5 ± 1.8 | 13.9 ± 1.75 ∗ | 12.78 ± 1.75 ‡ | 13.6 ± 1.8 | < 0.001 |
| Creatinine (mg/dL) | 1.2 ± 0.37 | 1.19 ± 0.33 | 1.33 ± 0.45 ‡ | 1.16 ± 0.36 | 0.004 |

∗ Group A vs. Group B (p < 0.05), † Group A vs. Group C (p < 0.05), ‡ Group B vs. Group C (p < 0.05).

**Table Supplements 3** Clinical characteristics of patients with secondary prophylaxis (n = 99)

|  | **Total study cohort (n = 99)** | **Group A = Benefit (n = 30)** | **Group B = No Benefit (n = 12)** | **Group C = Neutral (n = 57)** | **P value** |
| --- | --- | --- | --- | --- | --- |
| Age (years) | 66.3 ± 12.7 | 63.7 ± 13.4 ∗ | 73 ± 6.4 ‡ | 66.3 ± 13 | 0.092 |
| Women (♀), n (%) | 21 (21) | 6 (20) | 2 (17) | 13 (23) | 0.877 |
| **Indication for ICD** |  |  |  |  |  |
| Ventricular fibrillation | 65 (66) | 21 (70) | 5 (42) | 39 (68) | 0.173 |
| Sustained ventricular tachycardia | 34 (34) | 9 (30) | 7 (58) | 18 | 0.173 |
| Cardiac resynchronisation therapy, n (%) | 7 (7) | 2 (7) | 0 (0) | 5 (9) | 0.557 |
| Generator replacement, n (%) | 29 (29) | 6 (20) | 4 (33) | 19 (33) | 0.408 |
| Inadequate ICD shock, n (%) | 8 (8) | 3 (10) | 1 (8) | 4 (7) | 0.888 |
| **Medical history** |  |  |  |  |  |
| Hypertension, n (%) | 70 (71) | 20 (67) | 8 (67) | 42 (74) | 0.750 |
| Dyslipidemia, n (%) | 42 (42) | 12 (40) | 6 (50) | 24 (42) | 0.837 |
| Diabetes mellitus, n (%) | 27 (27) | 5 (17) ∗ | 8 (67) ‡ | 14 (25) | 0.004 |
| Coronary artery disease, n (%) | 62 (63) | 17 (57) | 8 (67) | 37 (65) | 0.666 |
| Myocardial infarction, n (%) | 48 (48) | 12 (40) | 6 (50) | 30 (53) | 0.530 |
| Coronary artery bypass grafting, n (%) | 16 (16) | 5 (17) | 2 (17) | 9 (16) | 0.993 |
| Atrial fibrillation, n (%) | 36 (36) | 13 (43) | 4 (33) | 19 (33) | 0.636 |
| Stroke and/or TIA, n (%) | 20 (20) | 6 (20) | 4 (33) | 10 (18) | 0.464 |
| Chronic obstructive lung disease, n (%) | 9 (9) | 4 (13) | 2 (17) | 3 (5) | 0.287 |
| Peripheral artery disease, n (%) | 20 (20) | 4 (13) ∗ | 6 (50) ‡ | 10 (18) | 0.021 |
| **Medication** |  |  |  |  |  |
| ACEI or ARB or ARNI, n (%) | 79 (80) | 26 (87) | 10 (83) | 43 (75) | 0.440 |
| Betablocker, n (%) | 86 (87) | 26 (87) | 11 (92) | 49 (86) | 0.868 |
| Loop diuretics, n (%) | 48 (48) | 12 (40) | 6 (50) | 30 (53) | 0.530 |
| Aldosterone antagonist, n (%) | 34 (34) | 8 (27) | 4 (33) | 22 (39) | 0.501 |
| Amiodarone, n (%) | 21 (21) | 5 (17) | 4 (33) | 12 (21) | 0.490 |

ICD, implantable cardioverter defibrillator; TIA, transient ischaemic attack; ACEI or ARB or ARNI, angiotensin converting enzyme inhibitor or angiotensin receptor blocker angiotensin receptor-neprilysin inhibitor.

∗ Group A vs. Group B (p < 0.05), † Group A vs. Group C (p < 0.05), ‡ Group B vs. Group C (p < 0.05).

**Table Supplements 4** Echocardiographic, electrocardiographic and laboratory characteristics of patients with secondary prophylaxis (n = 99)

|  | **Total study cohort (n = 99)** | **Group A = Benefit (n = 30)** | **Group B = No Benefit (n = 12)** | **Group C = Neutral (n = 57)** | **P value** |
| --- | --- | --- | --- | --- | --- |
| **Echocardiography** |  |  |  |  |  |
| Left atrial diameter (mm) | 41.5 ± 6.8 | 41.5 ± 5.53 | 43 ± 6.82 | 41.3 ± 7.5 | 0.915 |
| Left ventricular ejection fraction (%) | 42 ± 13.3 | 40.3 ± 13.4 | 41.9 ± 14.6 | 43 ± 13.2 | 0.685 |
| **Electrocardiography** |  |  |  |  |  |
| Heart rate (beats/min) | 74.5 ± 22.2 | 73 ± 19.5 | 77.3 ± 26.6 | 74.8 ± 23.2 | 0.854 |
| Sinus rhythm, n (%) | 78 (79) | 24 (80) | 7 (58) | 47 (82) | 0.280 |
| Left bundle branch block, n (%) | 15 (15) | 5 (17) | 0 (0) | 10 (18) | 0.294 |
| **Labor** |  |  |  |  |  |
| Hemoglobin (g/dL) | 12.9 ± 2 | 13 ± 2.26 | 12.4 ± 1.8 | 13 ± 2.01 | 0.509 |
| Creatinine (mg/dL) | 1.1 ± 0.4 | 1.0 ± 0.34 | 1.22 ± 0.3 | 1.13 ± 0.45 | 0.050 |
